# Multiaxial Fatigue Damage Parameter and Life Prediction without Any Additional Material Constants

**DOI:** 10.3390/ma10080923

**Published:** 2017-08-09

**Authors:** Zheng-Yong Yu, Shun-Peng Zhu, Qiang Liu, Yunhan Liu

**Affiliations:** 1 Center for System Reliability & Safety, University of Electronic Science and Technology of China, Chengdu 611731, China; yuzhengyongyong@126.com (Z.-Y.Y.); liu_qiang_uestc@163.com (Q.L.); liu15680405143@163.com (Y.L.); 2 Key Laboratory of Deep Earth Science and Engineering, Ministry of Education, Sichuan University, Chengdu 610065, China

**Keywords:** multiaxial fatigue, low cycle fatigue, critical plane, damage parameter, life prediction

## Abstract

Based on the critical plane approach, a simple and efficient multiaxial fatigue damage parameter with no additional material constants is proposed for life prediction under uniaxial/multiaxial proportional and/or non-proportional loadings for titanium alloy TC4 and nickel-based superalloy GH4169. Moreover, two modified Ince-Glinka fatigue damage parameters are put forward and evaluated under different load paths. Results show that the generalized strain amplitude model provides less accurate life predictions in the high cycle life regime and is better for life prediction in the low cycle life regime; however, the generalized strain energy model is relatively better for high cycle life prediction and is conservative for low cycle life prediction under multiaxial loadings. In addition, the Fatemi–Socie model is introduced for model comparison and its additional material parameter *k* is found to not be a constant and its usage is discussed. Finally, model comparison and prediction error analysis are used to illustrate the superiority of the proposed damage parameter in multiaxial fatigue life prediction of the two aviation alloys under various loadings.

## 1. Introduction

Hot engine section components such as turbine discs and blades are often subjected to complex multiaxial cyclic loads, and their life evaluation is of great importance for ensuring the reliability and structural integrity of aero engines [1,2,3,4]. In particular, one or more of the following failure mechanisms threaten the integrity of these components: (a) multiaxial fatigue; (b) creep; (c) high temperature corrosion [5,6,7,8,9,10,11,12]. Multiaxial fatigue failure is one of the main failure modes of these components and has been widely studied. However, when compared with uniaxial fatigue, multiaxial fatigue presents additional challenges including additional hardening, and effects stemming from normal and shear mean stress. Due to the complexity of failure mechanisms in multiaxial fatigue and its dependence on microstructures, there are currently no widely accepted fatigue models used to predict multiaxial fatigue failures [13,14,15,16,17]. According to previous studies, the multiaxial fatigue life prediction models can be summed up into the following categories: the equivalent stress/strain models, energy-based criteria, damage mechanics-based models [18,19,20,21] and critical plane approaches [22,23,24]. The critical plane approach is regarded as the most promising framework according to its satisfactory prediction of fatigue life and crack failure direction and strong adaptability for materials or engineering applications [24].

At present, various multiaxial fatigue models have been developed based on the critical plane approach. These include the Fatemi–Socie (FS) model [25], the Wang–Brown (WB) model [26,27], and the Findley model [28]. However, most of these include one or more additional material constants, which were usually obtained from fitting fatigue data or the formula of material constants. It is very inconvenient to fit fatigue data and formulas for material constants of different models, especially under limited fatigue data conditions. Moreover, these material constants vary with the number of cycles to failure, and bring additional research on how to determine these material constants by testing [24]. Therefore, it is necessary to develop a simple and efficient multiaxial fatigue life prediction damage parameter without any additional material constants that are applicable for a variety of metal materials and loading conditions. Most energy-based models do not contain additional material constants, such as the Smith–Watson–Topper (SWT) parameter [29], Liu’s [30] and Chu’s model [31] etc. However, under pure torsion and torsion-dominated multiaxial loadings, the SWT model has demonstrated less accurate fatigue life predictions [23]. Liu’s model has two forms considering two crack failure modes—tensile cracking failure mode, and shear crack failure mode that is generally determined by fatigue tests [24,32]. Chu’s model averages the contribution from tensile behavior and shear behavior and provides an average crack direction of 22.5° as the critical plane [24,33]. This cannot really reflect the multiaxial fatigue of various load paths. FS mode has been widely recognized to have an accurate prediction of fatigue life and crack initiation direction for a wide variety of materials and under different loading conditions [34,35]. Ince and Glinka [36] present two multiaxial fatigue damage parameters on the maximum damage parameter plane, which characterize the fatigue behavior under proportional/non-proportional loadings and reflect the mean stress effect. However, the damage parameters of Ince–Glinka are divided into uniaxial and multiaxial forms and are not concise. Although expansive literature regarding multiaxial fatigue exists, the complex multiaxial fatigue failure of different materials remains elusive. Further research on accurate evaluation of fatigue life and crack failure is needed under multiaxial fatigue failure analysis.

The primary focus of the current work is to present a simple and efficient multiaxial fatigue damage parameter that is able to predict fatigue life under various load paths. The rest of the paper is structured as follows. In Section 2, several multiaxial fatigue models referenced in this paper are introduced briefly. Section 3 elaborates on two modified generalised strain energy/amplitude models, and then proposes a simple multiaxial fatigue damage parameter. Section 4 describes the fatigue test data under various load paths and test details for TC4 and GH4169. Section 4 also discusses the usage of additional material constant *k* of the FS model, while providing model validations and comparisons under symmetric and asymmetric multiaxial loadings. Section 5 contains a summary of current research in this paper.

## 2. Multiaxial Fatigue Damage Parameters

### 2.1. Fatemi–Socie Model

Based on physical failure observation of various metal materials and the Brown–Miller model, Fatemi and Socie [25] introduced a concept of equivalent shear strain amplitude, which reflects the effects of mean stress and additional hardening. This is achieved by substituting the normal strain amplitude for maximum normal stress on the critical plane. The critical plane of the FS model is usually considered to be the maximum shear strain plane [24,37,38]. Fatemi et al. [39] found that failure cracks of wrought specimens occur on or near to the orientation of the maximum shear strain plane. Yu et al. [40] performed an evaluation of the FS model with both the maximum shear strain plane and a maximum damage parameter plane. They found that treating the maximum damage parameter plane as the critical plane is optimal for the FS model. Jiang [41] discovered that the critical plane deviates from the maximum shear strain plane when the additional material constant *k* is not equal to zero. Therefore, the maximum damage parameter plane near the maximum shear strain plane is defined as the critical plane in this paper and the FS model is given as:

(1) $$\gamma_{a}\left(1+k\frac{\sigma_{n,max}}{\sigma_{y}}\right)=\frac{\tau_{f}^{\prime }}{G}{\left(2N_{f}\right)}^{b_{0}}+\gamma_{f}^{\prime }{\left(2N_{f}\right)}^{c_{0}}$$

where $\gamma_{a}$ and $\sigma_{n,max}$ are respectively the shear strain amplitude and maximum normal stress on the critical plane; $\sigma_{y}$ is the cyclic yield stress which can be obtained from 0.05% offset rule [40]; $\tau_{f}^{\prime }$ and $\gamma_{f}^{\prime }$ are the shear fatigue strength/ductility coefficients, respectively, $b_{0}$ and $c_{0}$ are the shear fatigue strength/ductility exponents;$\;N_{f}$ is the fatigue cycles to failure, *G* is the shear modulus, and *k* is the normal stress sensitivity coefficient which considers the influence of normal stress on crack propagation.

Note from [35] that the material coefficient *k* of the FS model is not a constant and it varies with the failure life, which is normally determined by:

(2) $$k=\left[\frac{\frac{\tau_{f}^{\prime }}{G}{\left(2N_{f}\right)}^{b_{0}}+\gamma_{f}^{\prime }{\left(2N_{f}\right)}^{c_{0}}}{\left(1+\nu_{e}\right)\frac{\sigma_{f}^{\prime }}{E}{\left(2N_{f}\right)}^{b}+\left(1+\nu_{p}\right)\varepsilon_{f}^{\prime }{\left(2N_{f}\right)}^{c}}-1\right]\frac{2\sigma_{y}}{\sigma_{f}^{\prime }{\left(2N_{f}\right)}^{b}}$$

where $\nu_{e}$ and $\nu_{p}$ are the elastic and plastic Poisson’s ratio, respectively; $\sigma_{f}^{\prime }$ and $\sigma_{f}^{\prime }$ are the fatigue strength/ductility coefficients, respectively; b and c are the fatigue strength/ductility exponents, respectively; E is the Young modulus.

### 2.2. Generalized Strain Energy/Amplitude (GSE/GSA) Damage Parameters

Considering the various advantages of previous models, Ince and Glinka [36] initially proposed a GSE damage parameter including shear and normal strain energy density from the viewpoint of strain energy. Referring to the critical plane of Chu [31,42], the maximum damage parameter plane is viewed as the critical plane of the GSE model, which is expressed as follows:

(3) $$GSE={\left(\tau_{max}\frac{{\Delta \gamma }_{e}}{2}+\frac{\Delta \tau }{2}\frac{{\Delta \gamma }_{p}}{2}+\sigma_{n,max}\frac{{\Delta \varepsilon }_{n,e}}{2}+\frac{{\Delta \sigma }_{n}}{2}\frac{{\Delta \varepsilon }_{n,p}}{2}\right)}_{max}=f\left(N_{f}\right)$$

where $∆\gamma_{e}/2$ and $∆\gamma_{p}/2\;$ are the elastic/plastic shear strain amplitude, respectively; $∆\varepsilon_{n,e}/2$ and $∆\varepsilon_{n,p}/2$ are the elastic/plastic normal strain amplitude, respectively; ∆τ/2 and $∆\sigma_{n}/2$ are the shear/normal stress amplitude, respectively; $\tau_{max}$ is the maximum shear stress. The damage parameter in Equation (3) considers the non-proportional hardening and mean stress effects by including the maximum shear stress and maximum normal stress on the critical plane.

Later, the contributions of all stress and strain components to fatigue damage are considered by Ince–Glinka [36]. The GSE damage parameter is then converted to GSA damage parameters which has the same advantages of GSE. The GSA damage parameter can be given as:

(4) $$GSA={\left(\frac{\tau_{max}}{\tau_{f}^{\prime }}\frac{∆\gamma_{e}}{2}+\frac{∆\gamma_{p}}{2}+\frac{\sigma_{n,max}}{\sigma_{f}^{\prime }}\frac{∆\varepsilon_{n,e}}{2}+\frac{∆\varepsilon_{n,p}}{2}\right)}_{max}=f\left(N_{f}\right)$$

Based on the fully-reversed uniaxial and torsion Manson–Coffin equation using the uniaxial fatigue properties of the material, the relationship between the damage parameter and life can be deduced as [33]:

(5) $$GSA=\left[\left(1+\nu_{e}\right)\frac{\sigma_{f}^{\prime }}{E}{\left(2N_{f}\right)}^{2b}+\left(1+\nu_{p}\right)\varepsilon_{f}^{\prime }{\left(2N_{f}\right)}^{c}\right]+\left[\frac{\left(1-\nu_{e}\right)}{2}\frac{\sigma_{f}^{\prime }}{E}{\left(2N_{f}\right)}^{2b}+\frac{\left(1-\nu_{p}\right)}{2}\varepsilon_{f}^{\prime }{\left(2N_{f}\right)}^{c}\right]$$

The GSA model with the critical plane of the maximum damage parameter considers the average contribution of normal strain and shear strain damage. The left part of the middle plus sign in Equation (5) corresponds to the shear strain damage and the right part corresponds to the normal strain damage. For pure shear fatigue and shear dominated multiaxial fatigue, the maximum damage parameter plane is near the maximum shear strain plane. This results in small normal strain damage, especially under surface strengthening conditions [43]. However, the existence of the right part of the middle plus sign in Equation (5) makes the predicted life extremely high, resulting in severe prediction errors. For uniaxial fatigue, Ince [33,36] provides the following equation:

(6) $$GSA_{axial}=\frac{\sigma_{max}}{\sigma_{f}^{\prime }}\frac{∆\gamma_{e}}{2}+\frac{∆\gamma_{p}}{2}=\frac{\sigma_{f}^{\prime }}{E}{\left(2N_{f}\right)}^{2b}+\varepsilon_{f}^{\prime }{\left(2N_{f}\right)}^{c}$$

Note from [33] that Equation (6) provides satisfactory life prediction under uniaxial fatigue loadings.

## 3. Proposed Multiaxial Fatigue Damage Parameter

Note from Equation (4) that the plastic strain in the multiaxial fatigue damage parameters of Ince–Glinka is not normalized by the corresponding stress correction factor. Considering the effects of plastic normal/shear strain on fatigue failure, a modified GSA (MGSA) damage parameter is proposed as follows:

(7) $$MGSA=\frac{\tau_{max}}{\tau_{f}^{\prime }}\frac{∆\gamma }{2}+\frac{\sigma_{n,max}}{\sigma_{f}^{\prime }}\frac{∆\varepsilon }{2}\;$$

For pure shear fatigue under fully reversed uniaxial cyclic loadings, the shear strain amplitude on the maximum shear strain plane can be given as:

(8) $$\frac{∆\gamma }{2}=\frac{\tau_{f}^{\prime }}{G}{\left(2N_{f}\right)}^{b_{0}}+\gamma_{f}^{\prime }{\left(2N_{f}\right)}^{c_{0}}$$

Based on the fully-reversed uniaxial and torsion Manson–Coffin equation, the maximum shear stress $\tau_{max}$ on the critical plane can be expressed as [36]:

(9) $$\tau_{max}=\frac{∆\tau }{2}=\tau_{f}^{\prime }{\left(2N_{f}\right)}^{b_{0}}$$

The maximum normal stress and normal strain amplitude on the maximum shear strain plane are approximately zero in the case of pure shear fatigue. By combining Equation (7) with Equation (9), the relationship between the MGSA damage parameter and fatigue life $N_{f}$ can be deduced as:

(10) $$MGSA=\frac{\tau_{max}}{\tau_{f}^{\prime }}\frac{∆\gamma }{2}+\frac{\sigma_{n,max}}{\sigma_{f}^{\prime }}\frac{∆\varepsilon }{2}=\frac{\tau_{f}^{\prime }}{G}{\left(2N_{f}\right)}^{2b_{0}}+\gamma_{f}^{\prime }{\left(2N_{f}\right)}^{c_{0}}$$

The shear/normal strain amplitude in the MGSA model including elastic and plastic normal/shear strain amplitude is normalized by the corresponding stress correction factor. This considers the contributions of the shear strain and the normal strain to the total strain damage, with the stress correction factors reflecting the effects of mean stress and additional hardening. 

Similarly, following the concept of strain energy by considering the total shear and normal strain energy, as well as the critical plane near maximum shear strain plane, according to the GSE damage parameter in Equation (3)—a modified GSE (MGSE) model can be expressed as:

(11) $$MGSE=\tau_{max}\frac{∆\gamma }{2}+\sigma_{n,max}\frac{∆\varepsilon }{2}=\frac{\tau_{f}^{\prime }}{G}{\left(2N_{f}\right)}^{2b_{0}}+\tau_{f}^{\prime }\gamma_{f}^{\prime }{\left(2N_{f}\right)}^{b_{0}+c_{0}}$$

The critical planes of the MGSA and MGSE model are close to the maximum shear strain plane. This is applicable for most ductile metal materials with shear cracking [39]. The shear strain/strain energy of MGSA/MGSE contributes to the most fatigue damage that characterizes the crack initiation. The normal strain or strain energy is taken as the additional fatigue damage to reflect the crack propagation.

As aforementioned, the highlights of GSA damage parameter proposed by Ince–Glinka are the normal stress correction factor, $\sigma_{n,max}/\sigma_{f}^{\prime }$ and shear stress correction factor, $\tau_{max}/\gamma_{f}^{\prime }$. These characterize the effects of mean stress of tension and torsion. However, only the normal/shear elastic strains are normalized by the normal/shear correction factor, and the normal/shear plastic strains are important when normalized by normal/shear correction factors when considering the effect of mean stress on the plastic normal/shear strain. MGSA and MGSE are the initial models proposed. Moreover, considering that the effects of normal strain on the critical plane are different from that of shear strain, the normalized method of normal and shear strain should be different. In the proposed fatigue model, introducing the cyclic yield stress normalizes the normal strain. This reflects the effect of normal strain on crack propagation, and then leads to a simple multiaxial fatigue damage parameter (DP):

(12) $$DP_{proposed}=\frac{\tau_{max}}{\tau_{f}^{\prime }}\frac{∆\gamma }{2}+\frac{2\sigma_{n,max}}{\sigma_{y}+\sigma_{f}^{\prime }}\frac{∆\varepsilon }{2}=\frac{\tau_{f}^{\prime }}{G}{\left(2N_{f}\right)}^{2b_{0}}+\gamma_{f}^{\prime }{\left(2N_{f}\right)}^{c_{0}}$$

The proposed damage parameter in Equation (12) differentiates the effects of shear strain and normal strain on fatigue damage on the critical plane near the maximum shear strain plane. This accurately describes the different behaviours that shear strain promotes crack initiation and normal strain accelerates crack propagation [40].

## 4. Experimental Validation and Model Comparison

### 4.1. Materials and Multiaxial Fatigue Data

In order to evaluate and validate the current models, uniaxial and multiaxial fatigue test data of aero engine alloys TC4 and GH4169 [23,44,45] are used for model comparison. For TC4, the solid specimens with 6 mm diameter/15 mm gauge length are for axial fatigue tests. The tubular specimens with 17 mm outside diameter, 14 mm inside diameter, and 32 mm gauge length are for multiaxial fatigue tests. Furthermore, the tubular specimens with 16 mm outside diameter, 12 mm inside diameter, and 50 mm gauge length are for fatigue tests of GH4169. More details of the specimen configuration and dimensions of TC4 and GH4169 are referenced in [23,44,45]. The fatigue tests of TC4 and GH4169 are respectively carried out at room temperature and 650 °C under different strain load paths. Monotonic and cyclic properties of TC4 and GH4169 are respectively listed in Table 1 and Table 2. Due to the limited fatigue data of GH4169 at 650 °C, its torsional properties can be crudely evaluated from uniaxial properties [40]:

(13) $$\tau_{f}^{\prime }=\frac{\sigma_{f}^{\prime }}{\sqrt{3}};\;\gamma_{f}^{\prime }=\sqrt{3}\varepsilon_{f}^{\prime };\;b_{0}=b;\;c_{0}=c;\;G=\frac{E}{2\left(1+\nu^{*}\right)}$$

All load paths include uniaxial, pure torsion, proportional, 45° and 90° non-proportional loadings under triangle/sine wave strain-controlled mode. The fatigue tests of TC4 were conducted under sine wave strain-controlled for symmetric and asymmetric loadings, which are respectively shown in Table 3 and Table 4. For GH4169, the specimen numbers marked with * are tested under sine wave loading and the others are tested under triangle wave loading in Table 5.

### 4.2. Discussion on the Additional Material Parameter of the FS Model

Although the FS model depicts a strong ability to predict fatigue life for various metal materials, its additional material parameter *k* is determined by different methods that can produce different results and lead to somewhat different life predictions. Commonly, material parameter *k* is obtained by fitting the uniaxial fatigue data against the pure torsion fatigue data [23,38,39,40,46,47,48]. Shamsaei et al. [49] found that the FS damage parameter is not sensitive to the *k* value and suggested that the *k* can be considered as 1 in the uniaxial form of FS model. However, Li et al. [46] indicated that the inappropriate value of *k* is a primary reason for the FS model’s inaccurate predictions. Therefore, it is necessary to evaluate the effective method for determining *k* in the FS model to make a better fatigue life correlation.

**Figure 1:**
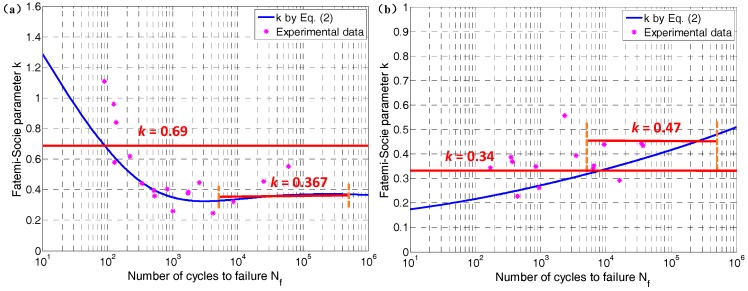
Fatemi–Socie parameter *k* for (**a**) TC4 and (**b**) GH4169.

The value of *k* has been evaluated to be 0.69 for TC4 and 0.34 for GH4169 by fitting uniaxial fatigue data in Table 3 and Table 5. This widely used method of fitting fatigue data to obtain *k* apparently has an acceptable prediction for most metal materials according to previous studies [23,38,39,40,46,47,48]. However, the changing trend of *k*, as the coefficient reflects the influence of normal stress on crack propagation, is different to the increase of $N_{f}$ for different materials according to Equation (2). The blue line in Figure 1 shows that *k* decreases with fatigue life for TC4 and increases with fatigue life for GH4169. The pink star scatter indicates the value of *k* calculated based on the uniaxial fatigue test data. As can be seen from Figure 1, the fitted *k* can indeed approximate the mean value of *k*, so the life prediction error of the current fatigue tests is the lowest. In addition, *k* can be obtained from Equation (2). However, fatigue life is found to display a greater sensitivity to the *k* value in the low cycle life regime, and is less sensitive to the *k* value in the high cycle life regime throughout repeated trial calculations. Therefore, another method of obtaining *k* is introduced in the current research. Considering the insensitivity of fatigue life to *k* in the high cycle life regime and the life prediction ability of the FS model, the mean value of *k* in the fatigue life range 5 × 10^3^ ≤ $N_{f}$ ≤ 5 × 10^4^ is considered as the model coefficient of the FS model according to [50]. Based on this method, the *k* is calculated to be 0.367 for TC4 and 0.47 for GH4169.

**Figure 2:**
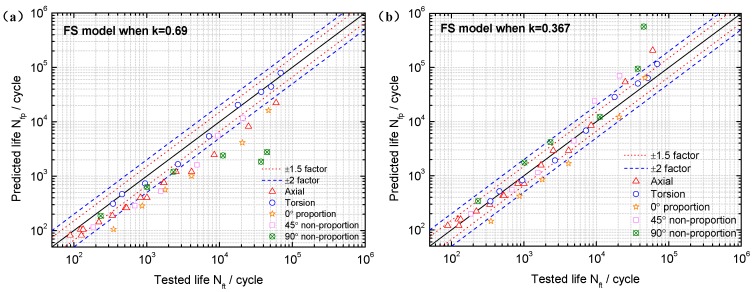
Comparison between tested lives and predicted lives using the Fatemi–Socie (FS) model when (**a**) *k* = 0.69 and (**b**) *k* = 0.367 for TC4 under symmetric loadings.

**Figure 3:**
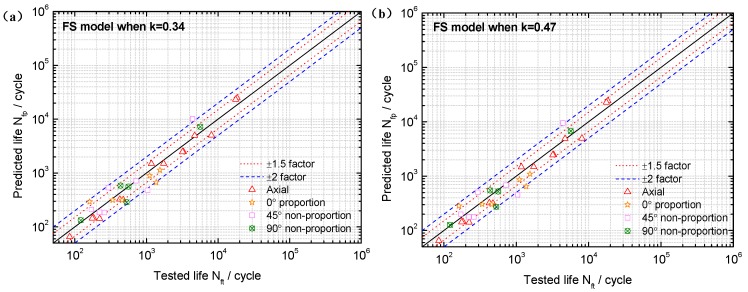
Comparison between tested lives and predicted lives using the FS model when (**a**) *k* = 0.34 and (**b**) *k* = 0.47 for GH4169.

Note from Figure 2 that the FS model gives a better fatigue life correlation when *k* = 0.367 than that when *k* = 0.69 for TC4 under symmetric loadings. Although the difference between the 0.367 and the *k* value estimated by the uniaxial fatigue test data is large, the FS model has a better fatigue life prediction ability for TC4, which further indicates that lower life is not sensitive to *k* values and higher life is more sensitive to *k* values. For GH4169, the FS model using the *k* obtained from the abovementioned two methods gives a good fatigue life correlation as shown in Figure 3. Therefore, the *k* value obtained from high cycle life regime based on Equation (2) can make the FS model have better life prediction ability and adaptability for different materials.

### 4.3. Model Validation and Comparison

**Figure 4:**
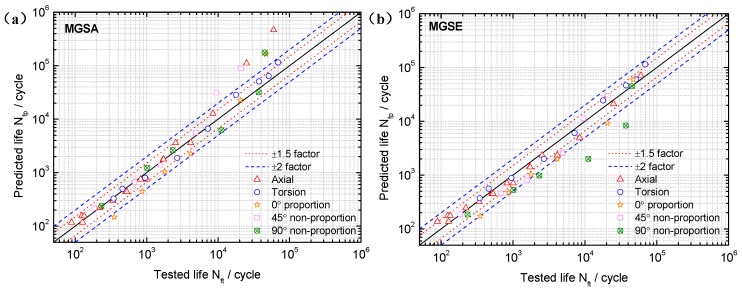
Comparison between tested lives and predicted lives using (**a**) the MGSA model (Equation (10)) and (**b**) the MGSE model (Equation (11)) for TC4 under symmetric loadings.

**Figure 5:**
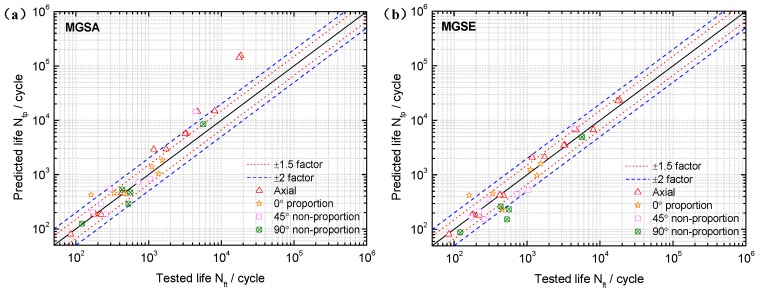
Comparison between tested lives and predicted lives using (**a**) the MGSA model (Equation (10)) and (**b**) the MGSE model (Equation (11)) for GH4169 at 650 °C.

**Figure 6:**
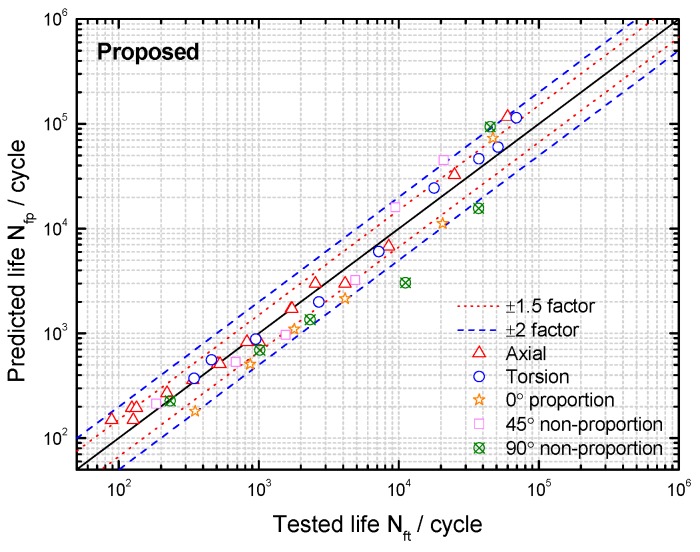
Comparison between tested lives and predicted lives using the proposed damage parameter for TC4 under symmetric loadings.

**Figure 7:**
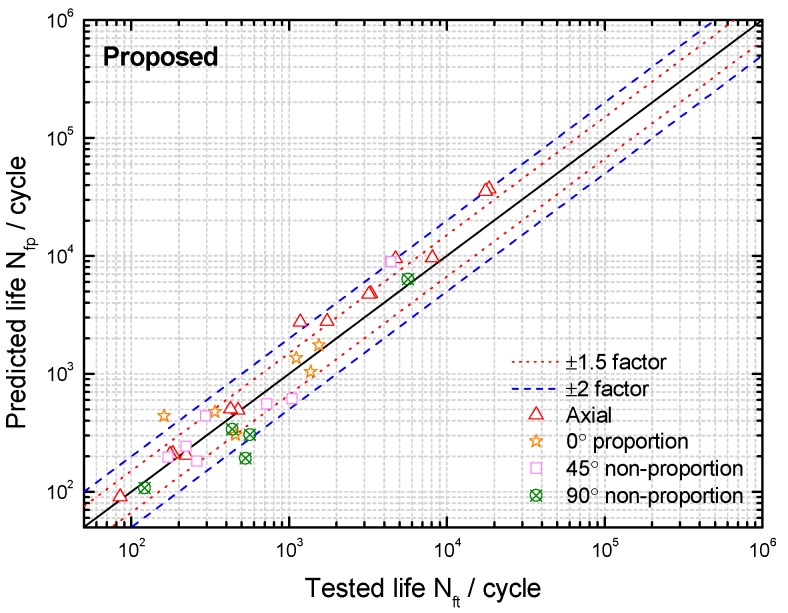
Comparison between tested lives and predicted lives using the proposed damage parameter for GH4169 at 650 °C.

Based on the limited fatigue test data of TC4 and GH4169, a model validation and comparison was performed in this section. Both the MGSA and MGSE model give an acceptable fatigue life correlation for TC4 and GH4169 under symmetric multiaxial loadings as shown in Figure 4 and Figure 5. However, the MGSA model overestimates fatigue life in the high cycle life regime and the life prediction of MGSE model is over-conservative for the multiaxial non-proportional loadings in the low cycle life regime. The proposed damage parameter overcomes the shortcomings of the above two modified damage parameters as the prediction results of TC4 and GH4169 in Figure 6 and Figure 7 show that the predicted fatigue lives in the high cycle life regime are all within or near the ±2 bands. Additionally, the predicted fatigue lives under multiaxial non-proportional loadings in the low cycle life regime are not particularly conservative when compared with the MGSE model. Thus, the proposed damage parameter in Equation (12) (termed as ′Proposed′ in the figures of this analysis) gives a better ability for multiaxial fatigue life prediction. 

**Figure 8:**
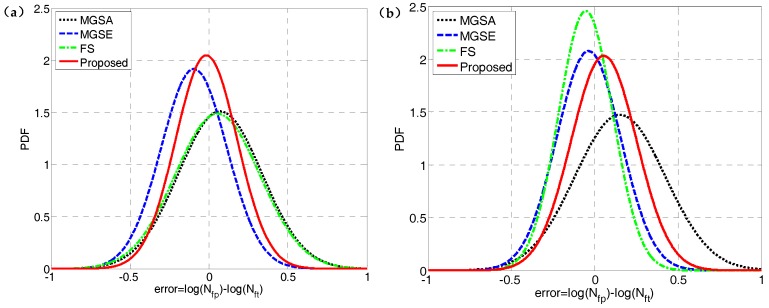
Probability density functions of model prediction errors for: (**a**) TC4 under symmetric loadings; and, (**b**) GH4169 at 650 °C.

The FS model is widely used throughout previous research and application due to its strong fatigue life prediction ability for various materials [46,47,48,50]. The prediction results of the FS model using the *k* obtained from the high cycle life regime in Section 4.2 are compared with those of the proposed damage parameter. The probability analysis is also introduced for model prediction error analysis of TC4 and GH4169 as shown in Figure 8. This shows that the proposed damage parameter provides more accurate predictions than previous models due to the lowest mean value and standard deviation of model prediction errors for TC4, as well as the fatigue life correlation of MGSE. For GH4169, the superiority of the FS model is reflected through its life prediction accuracy owing to its additional material parameter. However, both the proposed damage parameter and the GSE model provide reasonably accurate predictions as most of the predicted lives provided by them are within factors of two, as shown in Figure 5b and Figure 7.

**Figure 9:**
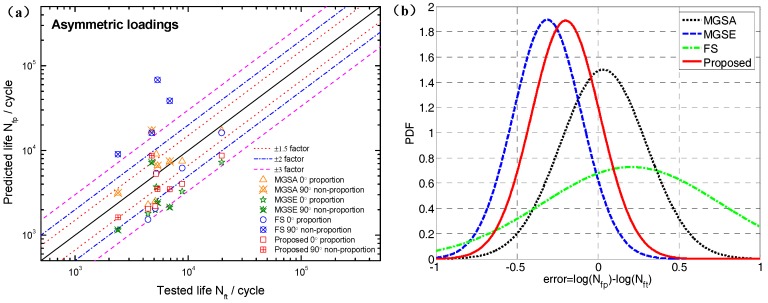
(**a**) Comparison between model predictions and tested fatigue lives of TC4 under asymmetric loading and (**b**) probability density functions of model prediction errors.

It is necessary to evaluate the fatigue life under asymmetric loadings because the mean stress effect is common in engineering components [51,52,53]. The abovementioned fatigue models claim to describe the effects of mean stress, so a multiaxial fatigue life prediction comparison under asymmetrical loadings is performed as shown in Figure 9. Figure 9 shows that nearly all of the predicted lives of the MGSE, MGSA and proposed damage parameter are within the factors of three. Additionally, some of the lives predicted by the FS model deviate greatly from the tested ones. Figure 9b shows that the proposed damage parameter gives the best prediction accuracy when compared with other models considering the low mean value and standard deviation of model prediction error according to their overall performance. The fatigue life correlations provided by MGSA and MGSE are more accurate than FS, which indicates that the normal stress correction factor, $\sigma_{n,max}/\sigma_{f}^{\prime }$ and the shear stress correction factor, $\tau_{max}/\gamma_{f}^{\prime }$ are advantageous for describing the mean stress effect. The reason why the FS model gives less accurate multiaxial fatigue life prediction under asymmetric loadings is that it cannot reflect the shear mean stress effect. However, the MGSA, MGSE and proposed damage parameter include the effect of normal mean stress and shear mean stress on the critical plane.

## 5. Conclusions

This paper attempts to put forward a simple and efficient multiaxial fatigue damage parameter without any additional material constants that can predict fatigue life under various load paths, and reflect the effects of normal/shear mean stress and non-proportional additional hardening. The FS model with an additional material parameter is widely recognised to have a good life prediction ability, which is introduced for model comparison. The conclusion of current studies is summarized below:

(1) The generalized strain amplitude/energy damage parameters are modified and the usage of these two damage parameters is simplified. The MGSA and MGSE damage parameters were elaborated upon by adding correction of normal/shear plastic strain amplitude by normal/shear stress correction factors based on GSA and GSE damage parameters.
(2) By considering the effects of normal strain and shear strain on the damage of the critical plane, the normal/shear strain is corrected by different forms of normal and shear stress correction factors. A simple and efficient multiaxial fatigue damage parameter is proposed based on the normal stress correction factor including cyclic yield stress.
(3) The material constant *k* of the FS model is discussed in this paper. It is found that the FS model using the fitted *k* provides less accurate fatigue life predictions for TC4, and a good performance of fatigue life prediction for GH4169 at 650 °C. However, when corresponding to the high cycle life regime, the mean value of *k* can enhance the fatigue life prediction ability of the FS model for the two materials. This indicates that fatigue life prediction results are more sensitive to the *k* in the low cycle life regime, and less sensitive to *k* in the high cycle life regime.
(4) The proposed MGSA and MGSE models provide acceptable fatigue life predictions for TC4 and GH4169 under various loadings. However, the MGSA model has shown poor performance in the high cycle life regime, and the MGSE model gives a conservative prediction for low-cycle non-proportional multiaxial loadings. The proposed damage parameter overcomes the shortcomings of the MGSA and MGSE models, and nearly all of its prediction results fall within a factor of ±2. It provides a better analysis for multiaxial fatigue life prediction under asymmetric loadings than others, while considering normal/shear mean stress effects. Also, further comprehensive assessment of the proposed damage parameter needs to be addressed for different materials under different loading paths.

## Figures and Tables

**Table 1:** Monotonic and fatigue properties of TC4 at room temperature.

Monotonic properties	E **(GPa)**	G **(GPa)**	$\sigma_{y}$ **(MPa)**	$\upsilon_{e}$	K **(MPa)**	***n***
	108.4	43.2	942.5	0.25	1054	0.0195
Uniaxial properties	$\sigma_{f}^{\prime }$ **(MPa)**	***b***	$\varepsilon_{f}^{\prime }$	***c***	$K^{\prime }$ **(MPa)**	$n^{\prime }$
	1116.9	− 0.049	0.579	− 0.679	1031	0.0478
Torsional properties	$\tau_{f}^{\prime }$ **(MPa)**	$b_{0}$	$\gamma_{f}^{\prime }$	$c_{0}$	$K_{0}^{\prime }$ **(MPa)**	$n_{0}^{\prime }$
	716.9	− 0.06	2.24	− 0.8	446.7	0.016
	716.9	− 0.06	2.24	− 0.8	446.7	0.016

**Table 2:** Material properties of GH4169.

*T* (°C)	*E* (GPa)	$\sigma_{y}$ (MPa)	$\sigma_{f}^{\prime }$ (MPa)	$\varepsilon_{f}^{\prime }$	*b*	*c*	$K^{\prime }$ (MPa)	$n^{\prime }$
650	182	626.4	1476	0.162	− 0.086	− 0.58	1933	0.1483

**Table 3:** Fatigue test data of TC4 for symmetric loadings.

No.	φ°	$\varepsilon_{a}$ (%)	$\gamma_{a}$ (%)	$N_{f}$ (Cycles)	No.	φ°	$\varepsilon_{a}$ (%)	$\gamma_{a}$ (%)	$N_{f}$ (Cycles)
1	\	0.55	\	60,048	23	\	\	1.302	2691
2	\	0.6	\	25,069	24	\	\	1.645	951
3	\	0.7	\	8457	25	\	\	1.942	459
4	\	0.8	\	4135	26	\	\	2.309	345
5	\	0.8	\	2544	27	0	0.345	0.648	47,195
6	\	0.9	\	1708	28	0	0.427	0.71	20,611
7	\	0.9	\	1730	29	0	0.576	0.938	4141
8	\	1.1	\	1007	30	0	0.687	1.111	1795
9	\	1.1	\	822	31	0	0.863	1.371	868
10	\	1.3	\	510	32	0	1.391	2.038	351
11	\	1.3	\	529	33	45	0.391	0.643	20,953
12	\	1.5	\	339	34	45	0.418	0.702	9478
13	\	1.7	\	221	35	45	0.496	0.831	4898
14	\	2	\	124	36	45	0.62	1.043	1563
15	\	2	\	134	37	45	0.772	1.255	683
16	\	2.3	\	89	38	45	1.224	1.756	185
17	\	2.3	\	127	39	90	0.349	0.639	45,138
18	\	\	0.798	69,269	40	90	0.418	0.704	37,273
19	\	\	0.833	51,146	41	90	0.499	0.821	11,152
20	\	\	0.848	37,449	42	90	0.556	0.934	2332
21	\	\	0.889	17,887	43	90	0.632	1.079	1017
22	\	\	1.038	7218	44	90	1.229	1.7	233

**Table 4:** Fatigue test data of TC4 for asymmetric loadings.

No.	φ°	$\varepsilon_{a}$ (%)	$\gamma_{a}$ (%)	$\varepsilon_{m}$ (%)	$\gamma_{m}$ (%)	$N_{f}$ (Cycles)
1	0	0.382	0.714	0	1.17	19,750
2	0	0.556	0.889	0	1.495	5126
3	90	0.485	0.828	0	1.409	4772
4	0	0.438	0.719	0.754	0	5225
5	0	0.565	0.911	1.042	0	4422
6	90	0.42	0.698	0.428	0	6878
7	90	0.502	0.822	0.974	0	2394
8	0	0.466	0.726	0.978	1.386	8867
9	90	0.423	0.705	0.826	1.253	5357

**Table 5:** Fatigue test data of GH4169 at 650 °C.

No.	φ°	$\varepsilon_{a}$ (%)	$\gamma_{a}$ (%)	$N_{f}$ (Cycles)	No.	φ°	$\varepsilon_{a}$ (%)	$\gamma_{a}$ (%)	$N_{f}$ (Cycles)
1	-	1.4855	\	85	17	0	0.408	0.592	1544
2	\	1.1035	\	223	18	45	0.524	0.745	722
3	\	1.093	\	183	19	45	0.553	0.813	295
4	\	1.0975	\	176	20	90	0.548	0.833	436
5	\	0.853	\	475	21	90	0.586	0.838	563
6	\	0.848	\	425	22	0	0.546	0.884	458
7	\	0.5975	\	1743	23	45	0.704	1.09	171
8	\	0.5985	\	1174	24	45	0.701	1.16	260
9	\	0.55	\	3290	25	90	0.783	1.33	121
10	\	0.5505	\	3204	26 *	0	0.54	0.896	338
11	\	0.5	\	8097	27 *	0	0.536	0.945	161
12	\	0.501	-	4732	28 *	0	0.427	0.633	1108
13	\	0.4285	\	18531	29 *	0	0.448	0.709	1370
14	\	0.4305	\	17633	30 *	45	0.478	0.749	1048
15	45	0.354	0.42	4420	31 *	45	0.625	1	222
16	90	0.397	0.479	5665	32 *	90	0.613	1.01	529

Note: The specimen number marked with * is under sine wave loading.

## Nomenclature

$\gamma_{a}$	Maximum shear strain amplitude on the critical plane
$\sigma_{n,max}$	Maximum normal stress on the critical plane
$\sigma_{y}$	Cyclic yield stress
$\tau_{f}^{\prime }$	Shear fatigue strength coefficient
$\gamma_{f}^{\prime }$	Shear fatigue ductility coefficient
$N_{f}$	Number of cycles to failure
G	Shear modulus
$b_{0}$	Shear fatigue strength exponent
$c_{0}$	Shear fatigue ductility exponent
k	Fatemi–Socie parameter
$v_{e}$	Elastic Poisson’s ratio
$v_{p}$	Plastic Poisson’s ratio
b	Fatigue strength exponent
c	Fatigue ductility exponent
$\sigma_{f}^{\prime }$	Fatigue strength coefficient
$\varepsilon_{f}^{\prime }$	Fatigue ductility coefficient
$∆\gamma_{e}$	Elastic shear strain range on the critical plane
$∆\gamma_{p}$	Plastic shear strain range on the critical plane
$\tau_{max}$	Maximum shear stress on the critical plane
$∆\varepsilon_{n}$	Normal strain range acting on the critical plane
$∆\varepsilon_{n,e}$	Elastic normal strain range on the critical plane
*E*	Young modulus
$v^{*}$	Effective Poisson’s ratio
$N_{ft}$	Experimental life
$N_{fp}$	Model predicted life
FS	Fatemi–Socie
GSA	Generalized strain amplitude
GSE	Generalized strain energy
MGSA	Modified generalized strain amplitude
MGSE	Modified generalized strain energy
